# Development of the Sensory–Motor Dysfunction Questionnaire and Pilot Reliability Testing

**DOI:** 10.3390/brainsci14060619

**Published:** 2024-06-20

**Authors:** Ushani Ambalavanar, Heidi Haavik, Nooshin Khobzi Rotondi, Bernadette Ann Murphy

**Affiliations:** 1 Faculty of Health Sciences, Institute of Technology, University of Ontario, 2000 Simcoe St. N., Oshawa, ON L1G 0C5, Canada; 2 Center of Chiropractic Research, New Zealand College of Chiropractic, Mount Wellington, Auckland 1060, New Zealand

**Keywords:** spine pain, sensorimotor integration, patient-reported outcome measure (PROM), test–retest reliability, internal consistency

## Abstract

Both chronic and recurrent spinal pain alter sensorimotor integration (SMI), which is demonstrated using complex neurophysiological techniques. Currently, there is no patient-reported outcome measure that documents and/or assesses SMI in populations with spinal problems. The purpose of this study was to develop the Sensory–Motor Dysfunction Questionnaire (SMD-Q) and assess its test–retest reliability and internal consistency in individuals with recurrent spinal pain. The SMD-Q was developed based on the existing literature on motor control disturbances associated with disordered SMI. The initial SMD-Q drafts underwent review by two separate panels of subject matter experts and a focus group with subclinical spine pain. Their suggestions were incorporated into the questionnaire prior to reliability testing. The questionnaire was administered twice at a seven-day interval using Qualtrics^TM^. A total of 20 participants (14 females and 6 males; 20.95 ± 2.46 years of age) completed the study. Quadratic weighted kappa (K_w_) was used to assess test–retest reliability and Cronbach’s alpha (α) was used to assess internal consistency. Four items had a K_w_ < 0.40, seven had a 0.40 < K_w_ < 0.75, and one had a K_w_ > 0.75 (excellent agreement), with excellent internal consistency (α > 0.90). The pilot SMD-Q appears to reliably measure altered SMI, suggesting that revisions and testing with a larger sample are worth pursuing.

## 1. Introduction

Musculoskeletal pain and/or injury to the vertebral column leads to plastic changes (e.g., negative cognitive, emotional, and autonomic responses) [1], resulting in minor changes in motor control strategies [2,3], to protect the injured area and promote healing. It has been found that the paraspinal muscles exhibit structural [1,4,5,6,7,8,9,10,11,12,13,14] and functional [4] changes in response to an initial injury or painful experience. However, if this persists for a long period of time, those altered motor control strategies can persist and become maladaptive [2,3], resulting in altered cortical reorganization [15,16] within the somatosensory, sensorimotor, and motor control cortices [17,18,19,20]. It has been proposed that altered sensory input from deep paraspinal tissues within the vertebral column gives rise to maladaptive central changes [21,22]. The altered proprioceptive input to the central nervous system (CNS) following the physiological changes within the paraspinal muscles in response to an injury has been proposed to induce maladaptive motor strategies and movement patterns/biomechanics by affecting neuromuscular function via the central feedforward and feedback control mechanisms [21]. This is supported by the functional connectivity literature, which found differences between the healthy participants and populations with chronic musculoskeletal pain in the threat-detecting salience network and between the salience network and the self-representational default mode network [1,12,13,14], which are thought to reflect the pain becoming an intrinsic part of the self-perception [1]. Thus, once injury or pain is present, it induces an ongoing cycle of maladaptive motor strategies that impact motor function, which may last beyond the healing of the original injury if left untreated. It appears that this functionality of deep paraspinal tissues plays a pivotal role in driving the development and chronicity of musculoskeletal spinal pain, i.e., acute to subclinical to chronic [21,22].

The maladaptive changes that occur in response to an injury or pain have been linked to body schema (i.e., internal body representation) and multisensory integration, as it facilitates altered sensorimotor integration (SMI) and multi-modal integration (MMI) [23] due to ongoing changes in sensory feedback from musculoskeletal pain and spinal problems [4,21,22]. SMI relies on an accurate internal body schema, which is dependent on the accurate awareness of sensory information about the environment (i.e., exteroception, such as visual, auditory, gustatory, smell, and touch) and internal processes within the body (i.e., interoception, such as proprioception, kinesthesia, pH, oxygen levels, etc.) to produce a task-specific motor response [24,25,26,27,28]. MMI is a natural part of SMI, which assimilates sensory inputs from different sensory modalities (e.g., sound and visual information) to modulate task-specific motor outputs [29,30]. The manifestations of altered SMI and MMI appear to be one of the maladaptive outcomes that occur in response to musculoskeletal pain related to spinal dysfunction, which include changes in central and cortical SMI [17,18,19], MMI [20], motor control [2,4], limb SMI and motor performance [31,32,33,34], and sensorimotor function [35,36,37,38,39,40,41,42,43].

As previously mentioned, the function of deep paraspinal muscles is a catalyst in chronic musculoskeletal pain development [21,22]. When neck pain persists beyond three months but the individual experiences intermittent rather than constant pain, it is referred to as subclinical spinal pain (SCSP). SCSP individuals have pain-free days and have not sought treatment [44,45] due to the recurrent nature of their pain. As a result, SCSP populations have been frequently utilized [44,46,47,48,49,50,51,52,53,54,55] to study how altered paraspinal afferent input may impact the CNS without the confounding effects of current pain [44,45], i.e., asymptomatic on the day of testing. This is important because acute pain is known to affect early somatosensory evoked potentials [56,57,58] and the presence of pain of any chronicity is also known to alter movement patterns [33,59]. Due to this, SCSP populations allow for the assessment of the longer-term neuroplastic changes in constructs of SMI and MMI, which may occur in response to ongoing changes in somatosensory input from the affected spinal region.

Individuals with SCSP have been shown to exhibit impairments in various aspects of SMI, such as disturbances in cognition (e.g., brain fog, reduced ability to remain focused for long periods of time, etc.) [60,61]; poor upper limb motor control (e.g., lifting/elevating) [54]; altered kinematic movement patterns (e.g., changes in the way you move) [62] during reaching [63], step down [64] or balance tasks [65,66]; reduced grip strength [67]; poor kinesthetic awareness of head and limbs (e.g., bumping head or limb into things) [45,51,55]; an impaired ability to learn and/or perform a novel motor task [46,48,52]; and a diminished ability to process both audio and visual information (e.g., mishearing, misreading, or misinterpreting) [47,49]. These controlled laboratory-based studies demonstrate altered sensorimotor and/or neuromuscular function in SCSP populations, which are well documented using complex neurophysiological techniques, such as electromyography, electroencephalography, trans-cranial magnetic stimulation, and somatosensory evoked potentials, etc. These controlled testing procedures are not feasible to execute in a clinical setting.

Elsig et al. [68] recommended the use of the craniocervical flexion test, assessments of laterality judgement accuracy, and movement control to objectively assess sensorimotor disturbances in those with SCSP in a clinical setting. Yet due to the recurrent nature of SCSP, these tests may not always detect the presence of maladaptive SMI and MMI, which are present once pain becomes chronic [21,22]. In order to document and/or quantify the subjective manifestations of altered SMI and MMI that might accompany recurrent or chronic spinal problems, a patient-reported outcome measure (PROM) is needed. Having a reliable PROM can alert clinicians to altered SMI and MMI, which may occur even when pain is not present.

There are various PROMs on sensory processing and/or integration; however, they are used for the assessment/diagnosis of individuals on the autism spectrum [69,70,71,72,73] or with suspected sensory disorders [72,74,75,76,77]. The PROMs on the subjective manifestations of pain in the cervical spine [78] and lumbar region [79,80,81] focus on the region of interest and/or how the pain impacts certain tasks of daily living at that moment or in the span of a few days. The PROMs that consider awareness and perception of pain focus on a region of the body [82,83,84,85] and fail to consider how disordered SMI due to musculoskeletal spinal dysfunction impacts various aspects of SMI. Thus, none of those PROMs allow for the quantification and/or documentation of the impact of altered somatosensory processing on SMI, which may accompany recurrent or chronic musculoskeletal dysfunction as a result of maladaptive plasticity arising from altered sensory feedback from dysfunctional areas of the spine.

The Sensory–Motor Dysfunction Questionnaire was developed to address these gaps and to examine the subjective aspects of disordered multimodal SMI in relation to musculoskeletal spinal problems. The primary purpose of this study was to describe the development of the questionnaire using constructs of SMI that have been found in spine pain populations. Given that SCSP populations present with a great degree of variability in deficits in constructs of SMI compared to acute or chronic spine pain populations, the constructs of SMI and MMI were administered to individuals with subclinical neck pain (more commonly found) to examine the test–retest reliability and internal consistency of this questionnaire. It is hypothesized that this questionnaire would be able to reliably capture the degree of altered SMI and MMI in those with recurrent vertebral column dysfunction.

## 2. Materials and Methods

This questionnaire development and reliability study was approved by the University of Ontario Institute of Technology’s Research Ethics Board (File #16317). This study was performed according to the principles set out by the Declaration of Helsinki for the use of humans in experimental research.

### 2.1. Phase 1: Conceptualization and Development of the Sensory–Motor Dysfunction Questionnaire

The Sensory–Motor Dysfunction Questionnaire (SMD-Q) was originally based on a review conducted by Haavik et al. [86] on vertebral column dysfunction and its impact on sensorimotor integration and motor control. The articles that were cited in that paper were also evaluated to formulate the items for the SMD-Q. The original draft of the SMD-Q consisted of 9 items. Each of these 9 items consisted of constructs of SMI, MMI, and motor control that have been documented in the literature and have also been shown to change following treatment of vertebral dysfunction. The items that were included pertained to balance [65,66], hand–eye coordination [41], head or full-body proprioception [45,51,55], reaching movement, grasping motion [67], motor performance [46,48,52,53], audio or visual processing, MMI [47,49], and motor performance during the processing of multi-modal stimuli [49].

The original draft of the SMD-Q posed negatively worded questions for all questions, except for the question on physical balance and hand–eye coordination, to gauge “how often they performed an action and/or completed a task” associated with the SMI-related constructs over the past week. A numeric rating scale with descriptors on either end of the scale (never for 0 and very frequently for 10) was used for each question, as shown in Appendix A.

### 2.2. Phase 2: Validity Testing with Expert Panel and Target Population

The first draft of the questionnaire was examined by two expert panels with subject matter expertise and 8 individuals (7 females and 1 male) with SCSP. They examined the relevance and comprehensiveness of the questions in the questionnaire, i.e., content and face validity.

Seven subject matter experts were invited to participate. These experts were clinician scientists in the realm of neurophysiology who worked at tertiary institutions as research academics and had published research relevant to the concept of altered SMI, MMI, and/or motor control in populations with spinal pain. Expert panel 1 included two chiropractor PhD academics and one physiotherapist PhD academic. Expert panel 2 consisted of two chiropractor PhD academics and two physiotherapist PhD academics.

The target population for the SMD-Q is individuals with SCSP; therefore, we invited individuals with SCSP from the local university population to provide feedback on whether the items posed are relevant to their spine pain. Eight individuals with SCSP attended an hour-long virtual one-on-one cognitive interview with the lead researcher after the completion of the questionnaire (i.e., retrospectively). The lead researcher used a verbal probing technique [87]. The types of questions asked by the researcher included enquiring about respondents’ thought processes when deciding to give a response; the level of difficulty in providing responses; what specific words within the questions meant to them; if there was any confusion regarding what was being asked in a given question; how responses may differ if a question was posed differently; if there are any other examples applicable for a given construct; clarity of instructions; and whether the questionnaire captured the symptoms that were relevant to their spine pain. The cognitive interview allowed the researcher to understand the respondents’ cognitive processes when responding to the questions and the relevance and clarity of the questions posed in the questionnaire.

#### 2.2.1. Examiner and Target Population Feedback

Expert panel 1 provided the following feedback: (1) recommendation to create/use a standardized set of clinical history questions about their spinal dysfunction, including questions such as intensity of pain; (2) provide concrete/real-world examples for the 9 items; (3) use of a visual analog scale with 5 descriptive anchors; and (4) improve the clarity of questions that require individuals to know their own normal state (i.e., balance and coordination). Expert panel 2 also recommended the inclusion of a standard set of clinical questions about their spinal dysfunction, such as location of pain, onset of pain, medical history, etc. Expert panel 2 also suggested (1) splitting a multi-itemed question (auditory or visual processing) into two separate items; (2) generating an item on cognition; and (3) providing relatable examples for all items.

An informal Delphi framework was used to obtain collective verbal agreement on the questions that were included in each section of the pilot version. All of the feedback was implemented but a unipolar scale with a descriptor on either end was used, as this yields higher reliability compared to bipolar scales [88] and is appropriate for the context in which the constructs are being assessed. Another item on lower limb coordination was included since upper limb coordination was being captured.

The feedback provided by the 8 SCSP participants during their cognitive interviews was analyzed qualitatively to produce a thematic schema (allowing for comparison across respondents) from the detailed summaries. The thematic schema concluded that there were no difficulties in the completion of the questionnaire as intended; however, different phrases or words should be implemented for the purpose of clarity.

#### 2.2.2. Pilot Version of the SMD-Q

The pilot version (post-feedback) of the SMD-Q consisted of 12 items measuring the frequency of poor motor performance or control while completing common daily activities of living that require SMI (including MMI) over the past week. This questionnaire allows for the determination of the degree of SMI, MMI, or motor performance problems for those with vertebral column dysfunction, which have been well documented in spine pain populations [21]. A unipolar scale with an end descriptor on either end was used for each question. “Never” was the anchor on the far left of the scale, and “nearly all the time” was the anchor on the far right of the scale. Each question was scored out of 100, similar to a visual analog scale. This questionnaire was designed with the intention that it can be administered to persons with chronic or recurrent spine pain to gauge the degree to which altered spinal function may be affecting a person’s central processing and, in turn, affecting functionality during activities of daily living.

Alongside the SMD-Q, a thirty-four-item standard clinical spinal dysfunction history form was created to enable alterations in the SMD-Q to be compared to a patient’s clinical background. This standard clinical spinal dysfunction history form contained 29 questions capturing the state of spinal symptomatology and 5 questions that examined the frequency of pain, intensity, and tolerability of pain, and poor posture over the past week. All of these questions are standard clinical questions that are normally discussed during a regular spinal assessment in a clinical setting (e.g., past injuries, headaches, neurological conditions, region of pain, onset of pain, duration of pain episodes, medication use, presence of other chronic diseases, etc.). This clinical spinal dysfunction history form was put together based on the two expert panels’ feedback to enable clinicians who want to monitor their patients’ potential MMI and SMI symptoms to also gather a set of standard clinical questions about their patient’s spinal dysfunction.

At the end of the questionnaire, participants were asked whether “anything happened in the past week (i.e., trip and/or fall, head injury, pain other than neck and/or back pain, change in stress levels or health and well-being, etc.) that might have caused you to answer this questionnaire differently than in an average week?”. This question was asked to gauge whether factors outside participants’ normal lifestyles impacted the consistency of their questionnaire responses.

**Figure 1:**
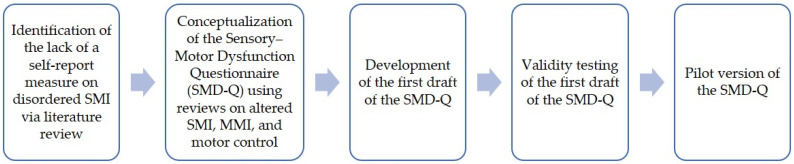
Development of the SMD-Q.

Figure 1 provides the development process of the SMD-Q from conceptualization to pilot version.

### 2.3. Phase 3: Reliability Testing of Pilot Version of the SMD-Q

#### 2.3.1. Participants

A total of 447 undergraduate students aged 18 to 35 and enrolled in courses offered by the Faculty of Health Sciences at Ontario Tech University were invited to participate in the online study via online course announcements. The participants were either in the second, third, or fourth year of their undergraduate studies. The students were eligible to participate if they were experiencing recurrent spine pain and/or stiffness for at least 3 months or more, i.e., a subclinical spinal pain (SCSP) participant. The eligible participants were required to not have any neurological conditions that are known to impact neural/cognitive function and/or neural processing (e.g., multiple sclerosis, stroke, Parkinson’s disease, and head injury with ongoing symptoms). The recruitment period was between 28 March and 22 April 2021. Twenty-five participants who fit the SCSP criterion were screened for eligibility (e.g., recurrent spinal pain, no treatment in the past month, etc.) and were assessed using the Von Korff Chronic Pain Grade scale. The Von Korff Chronic Pain Grade scale was used to determine the degree of pain-related disability and severity of the pain over the past 6 months [89,90]. Participants who had recurrent neck pain and scored between I and III on the Von Korff scale were deemed eligible (grade I (low disability–low intensity), grade II (low disability–high intensity), and grade III (high disability–moderately limiting). Individuals with a grade IV score were not eligible.

A total of 20 (14 F and 6 M) out of the 25 SCNP participants were eligible and completed the entirety of the study. The data collection began on 28 March and ended on 14 May 2021. The participants were not to have been treated or experienced an event that might have impaired their neural function and/or processing before completing the second administration of the questionnaire (e.g., head injury or illnesses that are known to impact central somatosensory processing, etc.). A pain visual analog scale was administered prior to starting the questionnaire to assess the level of neck pain on the day the questionnaire was completed. Participants provided informed consent electronically by selecting “I agree” upon reading the electronic consent form (presented before starting the questionnaire).

#### 2.3.2. Experimental Flow

**Figure 2:**
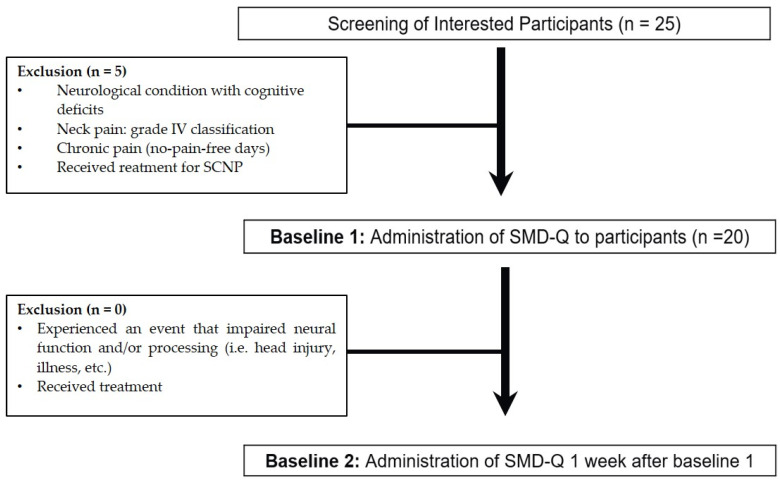
Experimental flow.

The SCNP participants completed the SMD-Q online using Qualtrics™ questionnaire administration software (https://www.qualtrics.com/blog/citing-qualtrics/, accessed on 18 June 2024) at baseline and 7 days later (see Figure 2).

### 2.4. Data Analysis

The slider questions used unipolar scales with fixed reference labels on either end of the horizontal scale, with the zero point on the far left and 100 on the far right labelled as never and nearly all the time, respectively. The length of the slider was approximately 400 pixels (~100 mm) on Qualtrics™. Instructions were provided and required participants to move their cursor to indicate their response (i.e., where they felt it best described them) for each question. The Qualtrics™ software quantified the location of the participant’s cursor on the sliding scale, which was a value between 0 (far left end) and 100 (far right end). If data were missing from one of the questions at either administration, they were removed from statistical analysis.

### 2.5. Statistical Analysis

All 20 datasets were included. All analyses were performed using IBM SPSS Statistics version 27 [91]. Descriptive statistics and the Shapiro–Wilk test were used to examine the distribution of the continuous variable data at baseline, revealing a non-normal distribution for all questions.

The weighted kappa (K_w_) statistic, which is the non-parametric equivalent to the intraclass correlation coefficient, was used to assess the intra-rater reliability of each question [92,93]. Four categories were created based on the distribution of the data via visual binning [91] to compute the weighted kappa statistics and 95% confidence intervals (CIs) for each question [94]. This categorization was ideal for a unipolar scale, as it is suggested to improve reliability [88,95]. The distribution of responses (frequencies) was also assessed based on (1) never/rarely; (2) occasionally; (3) often; and (4) almost always. The interpretation of the K_w_ value by Fleiss et al. [96] was used. A K_w_ ≤ 0.40 was considered poor agreement [96]. A 0.40 < K_w_ < 0.75 was considered fair to good agreement, and K_w_ ≥ 0.75 was considered excellent agreement [96].

The questionnaire was also assessed for internal consistency using Cronbach’s alpha, both at baseline and second administration. Internal consistency assesses the extent to which the items measure the same construct; it is an index of reliability [97]. A tiered Cronbach’s alpha was used to interpret the Cronbach’s alpha value as suggested by George et al. [98]; see Table 1 for cut-off values and interpretations. A Cronbach’s alpha value between 0.6 and 0.7 was considered and used as acceptable for this study, as this range is appropriate for scale development [99,100].

## 3. Results

A total of 20 participants (14 F and 6 M) completed the questionnaire at baseline and 7 days later. All descriptive data are reported as mean ± standard deviation. The average age of the 14 females was 20.71 ± 2.05 years and 21.50 ± 3.62 years for the 6 males.

None of the participants reported taking any medication for their pain and none reported having a confounding chronic disease. In response to the question asked at the end of the questionnaire about whether “anything happened in the past week (i.e., trip and/or fall, head injury, pain other than neck and/or back pain, change in stress levels or health and well-being, etc.) that might have caused you to answer this questionnaire differently than in an average week?”, three participants at both administrations and two participants at the second administration indicated that they had had a stressful week. When comparing their first and second questionnaire responses, they answered in a similar manner at each administration. Therefore, their data were not excluded from the reliability analysis.

Additional information was collected along with the SMD-Q, and the Qualtrics™ software did not allow us to extract timing for just the SMD-Q completion; therefore, an exact time to complete just the SMD-Q cannot be provided. However, the SMD-Q took 5 to 8 min to complete during piloting.

### 3.1. Spinal Characteristics

The spine pain characteristics of the sample are provided in Table 2.

### 3.2. Test–Retest Reliability and Internal Consistency of the SMD-Q

Questions 1, 2, 6, and 10, with values of K_w_ ≤ 0.40, are considered to have poor agreement [96]. While Questions 3, 4, 5, 7, 8, 9, and 12, with values of 0.40 < K_w_ < 0.75, are considered to have fair to good agreement [96]. Finally, Question 11, with values of K_w_ ≥ 0.75, is considered to have excellent agreement [96]. The K_w_ values for test–retest reliability are provided in Table 3.

The Cronbach’s alpha at baseline and 7 days later was 0.909 and 0.905, respectively. The SMD-Q has excellent internal consistency at the two time points.

## 4. Discussion

The SMD-Q is the first self-report measure that documents the degree of disordered SMI and MMI in individuals with recurrent spinal problems such as stiffness, ache, pain, or tension. This proof of concept and reliability study demonstrated that the item on neural processing during the bombardment of multiple sensory stimuli (Question 11) had excellent agreement. Four items had poor agreement. The SMD-Q had excellent internal consistency in both administrations.

### 4.1. Recommendations for the Next Version of the SMD-Q

In response to the findings of this pilot reliability testing, we recommend the following changes be made for items within the SMD-Q. Specifically, we describe recommendations on how the responses should be posed, calculated, and interpreted for the next iteration.

#### 4.1.1. Items

Questions with K_w_ ≤ 0.40 should either be rephrased or removed. Questions with fair to good agreement could be reworded for clarity and include more concrete examples that are consistent and relatable to the general public.

The low-weighted kappa statistic of Question 1 “Over the past week, how often have you had difficulty organizing everyday life routines, carrying out plans, or having difficulty performing familiar tasks in a logical manner?” could be the result of ambiguity. This question is intended to ask about brain fog, which is the lack of mental clarity when performing cognitive tasks [101]. This question will be removed as Question 1 in Appendix B sought to examine brain fog.

The lack of appropriate examples and clarity could have accounted for the poorly weighted kappa statistic for Question 2. This question required individuals to assess their physical balance in the past week by comparing it to their own healthy/usual balance, which could have led to confusion as there is no set recall period for healthy balance and the self-evaluation of balance is implicit. We suggest that the phrase “relative to what you consider your own healthy/usual balance” be removed. The question should be changed to “Over the past week, on average, how often did you have problems with your physical balance (i.e., frequent loss of balance or unsteadiness or feel like you might fall while walking, running or standing still, etc.)?”. Participants with altered balance are likely to have had it for longer than just one week, so they may not have a good comparison for what their usual balance is.

Question 6 “Over the past week, how often have you failed when you tried to pick something up or dropped things that you can normally do with ease?” yielded a low-weighted kappa statistic, possibly due to confusion from unclear phrasing. The failure to pick something up is similar to dropping things as they go hand in hand. This question was attempting to capture the grasping portion when completing a reach-and-grab motor task. This question could be rephrased to state “Over the past week, how often did you fail to pick something up that you initially dropped (i.e., slipped out of your hands or butter fingers, etc.)?” or it could be removed, as it may be capturing the same construct as Question 5.

Question 7 “Over the past week, how often have you had trouble recognizing objects that you normally recognize very quickly (e.g., recognizing that a cat ran past as opposed to just a fast-moving object)?” had a low-weighted kappa statistic. This may be the result of a lack of relatable examples and/or how infrequently this happens. We propose the implementation of the words “familiar” and “places or people” before and after “objects”, respectively. The following examples could be implemented: recognizing familiar face(s) when you walk past them at the grocery store, in the hallways at work or school; recognizing the place you frequently pass by, etc. This broadens the question to capture the construct of disordered processing of visual stimuli.

While a number of questions had fair to good agreement, we expect the frequencies for some of the questions may have been higher if not for the context in which the study took place (the COVID-19 pandemic and widespread lockdown in Ontario). Participants may not have been physically active or going outdoors and, as a result, some of the examples may not have applied to them. Question 4 will be broken down into two questions to allow for the assessment of head and neck proprioception and fully-body coordination (excluding head and neck) separately. See Appendix B for an updated questionnaire, specifically questions with more relatable examples for items that scored 0.4 < K_w_ < 0.75 (Questions 3–5, 7–9, and 12).

#### 4.1.2. Response

Response options should also be revised to categorical scales, which could improve the reliability of the questions alongside the proposed revisions. Based on the distributions from this first questionnaire, the four categories and the order they should be presented are as follows: (0) never/rarely occurs when doing this action/task (<1 day); (1) some or little of time when doing this action/task (1–2 days of the week); (2) often or a moderate amount of time when doing this action/task (3–4 days of the week); and (3) most or all of the time when doing this action/task (5 or more days of the week). The response options and frequency descriptors were drawn from the Center for Epidemiological Studies Depression Scale (CES-D), which is a validated tool to assess depression-related constructs over the past week [102]. The next version of the questionnaire could also include a total score of the questionnaire. Scores of 0, 1, 2, and 3 would be allocated to never/rarely, some or little of the time, often or a moderate amount of the time, and most or all of the time categories, respectively. The total score would be the summation of the score for each question in each subsection. The highest score that could be attained would be 36 (12 questions × 3 (highest score)) given that Question 1 will be removed and Question 4 will be split into two questions. A higher score would be suggestive of a greater degree of dysfunction from altered central processing. Testing of future versions could also investigate whether categories of SMI dysfunction could be created based on percentages of the total score. This would require testing with larger and more diverse subclinical and clinical populations (i.e., mild dysfunction, mild to moderate dysfunction, high dysfunction, etc.) with scores compared to pain-free control participants.

### 4.2. Strengths of This Study

The strengths of this study were as follows: (1) a one-week recall period; (2) a representative SCSP sample; and (3) no missing data between administrations. A recall period of one week was used, which has also been used in past research with other questionnaires [102]. The recall period is dependent on the characteristic of the construct of interest and the purpose of the assessment [103], which is the impact of disordered SMI and MMI processing on functionality during activities of daily living. This type of phenomenon has not been assessed in the literature using self-report measures; however, a phenomenon that changes rapidly in a short period of time requires a shorter recall period to effectively capture these data and minimize recall error [103]. The sample had a higher proportion of females as well as a range of individuals with SCSP that varied in chronicity and intensity, which is a fair representation of the SCSP population as indicated by the extensive literature on musculoskeletal pain [104,105,106].

### 4.3. Limitations of This Study

A limitation of this study was the small sample size and narrow age range (participants were all under 35 years of age); therefore, the findings from this study are not generalizable. A minimum sample size of 30 [107] or 32 [108] is recommended to obtain a more accurate estimate of agreement [109] for ordinal data with four categories. Given that this is the pilot of the SMD-Q, the weighted kappa values acquired from the sample of 20 (60%; out of a minimum of 30) are sufficient to guide the creation of the next version of the questionnaire, but not to confirm the findings in this study. It is also for this reason that exploratory factor analysis cannot be performed at this time since a sample size of 50 is the minimum acceptable threshold for a scale with less than 20 items [110,111,112]. As this study is focused on the development of the items, ceiling and floor effects could not be assessed as they are applicable to scores and scale responsiveness [113,114]. As this is the first study to assess reliability in an SCSP population, some items had a frequency of zero; however, it is expected that the frequency in the fourth category will (almost always) be greater when this questionnaire is administered to individuals with greater levels of spine dysfunction (in terms of chronicity and severity). Individuals over the age of 35 experience age-related declines in motor function [115,116] as well as deficits in constructs of SMI [117,118,119,120]. The next study should therefore test older participants once a revised version of the questionnaire has been created.

### 4.4. Future Directions

The SMD-Q needs further revisions and should be re-assessed for intra-rater reliability and internal consistency with a larger sample size (*n* > 32), including older participants, to confirm findings. Subsequent studies should also assess for exploratory factor analysis and ceiling and floor effects, followed by testing in other clinical populations. Future research could implement the SMD-Q in laboratory and clinical settings as a screening tool for altered SMI.

## 5. Conclusions

The pilot version of the SMD-Q has the potential to reliably assess constructs of SMI and MMI in individuals with SCSP. This novel questionnaire appears to capture dysfunctionality in constructs associated with SMI and MMI that occur within the span of a week. However, further revisions are needed to confirm this finding. The development of a reliable tool to assess constructs of SMI will enable clinicians to determine the onset of sensorimotor dysfunction and change an individual’s treatment plan to improve the constructs that suggest greater dysfunction or mild dysfunction. The SMD-Q could possibly be used as a screening tool for the quantification of self-reported altered SMI in laboratory and clinical settings.

## Figures and Tables

**Table 1:** Cronbach’s alpha cut-off values and interpretations [98].

Cut-Off Value	Interpretation
α < 0.5	Unacceptable
0.5 ≤ α < 0.6	Poor
0.6 ≤ α < 0.7	Questionable
0.7 ≤ α < 0.8	Acceptable
0.8 ≤ α < 0.9	Good
α ≥ 0.9	Excellent

**Table 2:** Spinal pain characteristics of the sample.

	SCNP
Duration/onset of SCSP (months)	24.25 ± 24.86
Pain VAS (cm)	
Baseline	19.45 ± 19.21
Follow-up	23.97 ± 24.76
Von Korff Chronic Pain Grade	
Score	1.5 ± 1.38
Disability points	0.9 ± 0.6
	***n***
Grade of Pain	
Grade 0	0
Grade I	11
Grade II	8
Grade III	1
Grade IV	0
**Clinical Characteristics**	***n***
Region of Spinal Pain	
Neck	20
Upper back	16
Mid back	16
Low back	16
Buttock	16

**Table 3:** Questions of the SMD-Q and the corresponding weighted kappa statistics and confidence intervals.

Constructs of Questions *	K_w_	95% CI
Question 1: Difficulty with higher-level thinking	0.189	(−0.272, 0.65)
Question 10: Uni-sensory processing of visual stimuli	0.3	(−0.121, 0.721)
Question 2: Physical balance	0.344	(−0.006, 0.695)
Question 6: Failed during grasping motor tasks	0.368	(−0.048, 0.785)
Question 3: Hand–eye coordination	0.5	(0.24, 0.76)
Question 4: Bumped into things or hit your head accidentally	0.567	(0.361, 0.772)
Question 5: Missed when using upper or lower limb	0.545	(0.149, 0.942)
Question 7: Misstepped	0.404	(−0.043, 0.85)
Question 8: Motor performance	0.444	(0.011, 0.878)
Question 9: Uni-sensory processing of auditory stimuli	0.545	(0.154, 0.937)
Question 12: MMI	0.587	(0.199, 0.976)
Question 11: Bombardment of sensory stimuli	0.798	(0.605, 0.991)

* Questions are presented and ordered according to K_w_ cut-offs.

## Data Availability

The data can be made available by the corresponding author upon reasonable request. The data are not publicly available due to ethical and privacy considerations.

## Appendix A. The Sensory-Motor Dysfunction Questionnaire

The following questionnaire has been designed to find out to what degree your spinal dysfunction is affecting your brain’s processing of sensory information and therefore affecting your function. Please answer **ALL** the scales and mark the **ONE** number on **EACH** scale that describes how you feel.

Question 1

Over the past week, how has your balance been?

0	1	2	3	4	5	6	7	8	9	10
Very Balanced									Very Unsteady	

Question 2

Over the past week, how has your hand-eye coordination been?

0	1	2	3	4	5	6	7	8	9	10
Very Coordinated									Clumsy and uncoordinated	

Question 3

Over the past week, how often have you bumped into things, or hit your head or elbow against objects accidently?

0	1	2	3	4	5	6	7	8	9	10
Never									Very Frequently	

Question 4

Over the past week, how often have you missed when you reached for an object (e.g., water bottle, kitchen tools, cup, pen, etc)?

0	1	2	3	4	5	6	7	8	9	10
Never									Very Frequently	

Question 5

Over the past week, how often have you missed when you pick something up and/or dropped things?

0	1	2	3	4	5	6	7	8	9	10
Never									Very Frequently	

Question 6

Over the past week, have you been struggling to perform a movement you normally do (e.g., hitting or catching the ball, tripped while walking or running, struggled with a musical instrument you normally play well, etc.)?

0	1	2	3	4	5	6	7	8	9	10
Never									Very Frequently	

Question 7

Over the past week, have you had trouble recognizing sounds or objects?

0	1	2	3	4	5	6	7	8	9	10
Never									Very Frequently	

Question 8

Over the past week, have you been having difficulties focusing when presented with multiple sensory “stimuli” (e.g., visual and auditory inputs)?

0	1	2	3	4	5	6	7	8	9	10
Never									Very Frequently	

Question 9

Over the past week, do you have difficulties responding in sensory rich environments that require you to pay attention to information from several different senses at once (i.e., driving, playing a sport, etc.)?

0	1	2	3	4	5	6	7	8	9	10
Never									Very Frequently	

## Appendix B. The Sensory-Motor Dysfunction Questionnaire (e.g., Questions about Your Brain-Body Communication)

This questionnaire seeks to determine to what degree your spinal dysfunction may be affecting how you process sensory information and therefore how it is affecting your function. ***Instructions:*** Please answer **ALL** the scales, by choosing the option that is most applicable to you, in the **past week**.

	**Never/Rarely When Doing This Task/Action (<1 Day)**	**Some or Little of the Time When Doing This Action/Task (1–2 Days)**	**Often or a Moderate Amount of Time When Doing This Action/Task (3–4 Days of the Week)**	**Most or All the Time When Doing This Action/Task (5 or More Days of the Week)**
**Question 1:** Over the past week, on average, how often did you have problems with your physical balance (i.e., frequent loss of balance or unsteadiness or feel like you might fall while walking, running or standing still, etc.)?	☐	☐	☐	☐
**Question 2:** Over the past week, on average, how often did you have hand-eye coordination problems (e.g., several typos when typing on your phone or computer keyboard, several mistakes when playing an instrument while reading sheet music, reading and miswriting, mis put key into door lock to unlock the door, making small mistakes when playing a sport with the hands, etc.)?	☐	☐	☐	☐
**Question 3:** Over the past week, how often have you accidentally bumped your head into things (i.e., hitting the top of your head when getting out of the car, bumping your head into a kitchen cupboard, etc.)?	☐	☐	☐	☐
**Question 4:** Over the past week, how often have you bumped into people or objects/things (i.e., table, wall, chair or leg of a chair, etc.) with other parts of your body?	☐	☐	☐	☐
**Question 5:** Over the past week, how often have you missed when you reached for an object without looking (e.g., phone, water bottle, cup, pen, book, kitchen tools, keys, etc.)?	☐	☐	☐	☐
**Question 6:** Over the past week, how often did you fail to pick something up that you initially dropped (i.e., slipped out of your hands or butter fingers, etc.)?	☐	☐	☐	☐
**Question 7:** Over the past week, how often have you missed when you had to use your leg or foot (i.e., misplaced or awkward step when walking or walking up/down the staircase or stepping up to a chair or stool, missed when kicking a ball or putting your foot in slip-on shoes, pushing against the gas pedal instead of the brake pedal, etc.)?	☐	☐	☐	☐
**Question 8:** Over the past week, how often have struggled to perform a movement you normally do well (e.g., missed when hitting or catching a ball, tripped while walking or running, struggled with a musical instrument you normally play well, pushing against the gas or brake pedal too hard or soft, biting the side of your mouth, lip or tongue while eating, etc.)?	☐	☐	☐	☐
**Question 9:** Over the past week, how often have you had trouble recognizing familiar sounds that you normally recognize very quickly (e.g., mishearing words or phrases when someone is speaking to you or mishearing lyrics of a familiar song)?	☐	☐	☐	☐
**Question 10:** Over the past week, how often have you had trouble recognizing familiar objects, places or people that you normally recognize very quickly (e.g., recognizing that a cat ran past as opposed to just a fast-moving object or recognizing familiar face(s) when you walk past them at the grocery store, in the hallways at work or school, or recognize the place that you frequently pass by, etc.)?	☐	☐	☐	☐
**Question 11:** Over the past week, how often have you been having difficulties concentrating in situations where there is a lot of background noise (e.g., people coughing, side conversations around you, driving while listening to music and passengers having a conversation with you, etc.)?	☐	☐	☐	☐
**Question 12:** Over the past week, how often have you had difficulties performing tasks that require you to combine information from more than one sense (sound, sight, smell, taste, etc.) at the same time (i.e., you aren’t as fast at combining sight and sound information from traffic so you have difficulty gauging how much time you have to cross the street, or during online gaming; or food is not smelling and tasting as it used to, or you are not as accurate at judging the space available to pass thorough an opening so you bump into things, etc.)?	☐	☐	☐	☐
